# A novel frameshift variant in *BCOR* causes congenital nuclear cataract

**DOI:** 10.1080/13816810.2024.2373248

**Published:** 2024-07-03

**Authors:** Vanita Berry, Manav B. Ponnekanti, Nikolas Pontikos, Roy A. Quinlan, Michel Michaelides

**Affiliations:** a UCL Institute of Ophthalmology, University College London, London, UK; b Moorfields Eye Hospital NHS Foundation Trust, London, UK; c UCL Medical School, University College London, London, UK; d Department of Biosciences, University of Durham, Durham, UK

**Keywords:** *BCOR*, X-linked dominant congenital nuclear cataract, WES

## Abstract

**Background:**

BCL6 co-repressor (*BCOR*) gene variants are involved in oculofaciocardiodental (OFCD) syndrome, acute myeloid leukaemia, renal tumours, and photoreceptor degenerative diseases. Here, we describe a British family with a pathogenic heterozygous variant in the *BCOR* gene causing congenital nuclear cataract.

**Methods:**

Whole-exome sequencing was conducted on an individual affected by X-linked dominant congenital cataract in a three-generation family to establish the underlying genetic basis. Bioinformatics analysis confirmed the variants with damaging pathogenicity scores.

**Results:**

A novel likely pathogenic frameshift variant *BCOR* NM_001123385.1: c.3621del; p.Lys1207AsnfsTer31, was identified and found to co-segregate with the disease in this family.

**Conclusions:**

This is apparently the first report of a variant in *BCOR* causing X-linked dominant congenital cataract which is potentially isolated or presenting with a remarkably mild systemic phenotype. Our findings extend the genetic basis for congenital cataract and add to the phenotypic spectrum of *BCOR* variants.

## 1. Introduction

The BCL6 corepressor (*BCOR*) is a transcription factor which plays a critical role in early embryogenesis, mesenchymal stem cell function, haematopoiesis, and lymphoid development. It is an X-linked gene (Xp11.4), with 15 exons and 17 splice variants. The most well-characterized phenotype for pathogenic *BCOR* variants is OFCD syndrome, a dominant disorder with wide-ranging systemic effects. These include facial abnormalities, such as cleft palate, cardiac septal, and valvular defects, various dental abnormalities, a spectrum of ocular defects, such as microphthalmia, anophthalmia, coloboma, and the focus of this paper is congenital cataract (1, 2).

Cataract, the opacification of the eye lens, can be a stand-alone phenotype or a part of a constellation of ocular defects. It is also associated with more than 200 syndromes (3–5). Congenital cataracts are clinically and genetically heterogeneous, displaying various phenotypes, such as nuclear, lamellar, posterior sub-capsule, cortical, and more. Congenital cataracts are usually detected at birth or during the first decade of life. They are mostly inherited as autosomal dominant traits, followed by recessive and X-linked inheritance (6, 7).

Around 57 congenital cataract-causing genes have been found to date, most of which are isolated cases. This group includes genes encoding lens soluble proteins including abg-crystallins; lens membrane proteins including connexins, aquaporins, and the receptor tyrosine kinase gene EPH receptor A2; an endoplasmic reticulum membrane-embedded protein, Wolframin; chromatin modifying protein-4B; cytoskeletal proteins including BFSP1 (filensin), BFSP2 (CP49, phakinin), vimentin; and finally, genes encoding transcription or developmental factors such as *EYA1, MAF, FOXE3, VSX2, PAX6, PITX3*, and *HSF4* (https://cat-map.wustl.edu/) (8, 9). Here, we report a likely pathogenic variant in *BCOR* that causes nuclear cataract in a three-generation family pedigree.

## 2. Material and methods

### 2.1. Phenotyping

**Figure 1.**
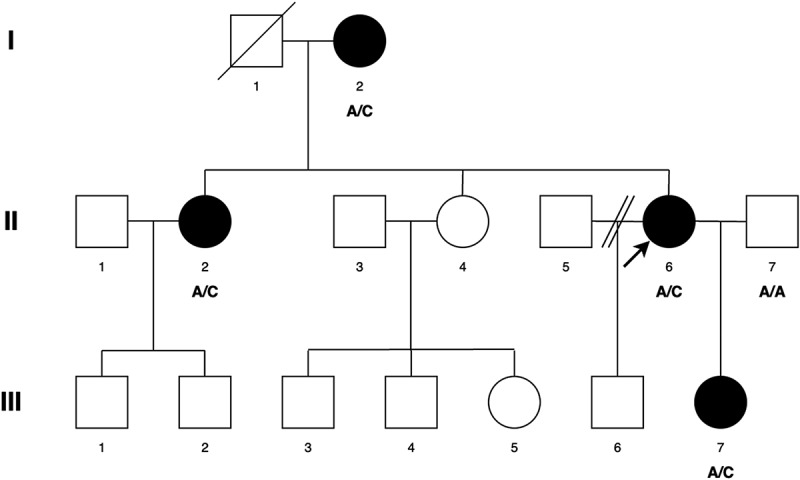
Abridged pedigree presenting with congenital cataract. Squares and circles symbolise males and females respectively. Diagonal lines through these symbols indicate deceased members of the pedigree. Open and filled symbols indicate unaffected and affected individuals respectively. Arrow indicates the proband (II-6). Affected individuals with the variant change at c.3621delA are labelled A/C (I-2, II-2, II-6, III-7). A/A is normal (II-7).

The patients in this pedigree were identified via the proband attending the Genetic Service at Moorfields Eye Hospital, London, UK. The study protocol adhered to the Tenets of the Declaration of Helsinki and was approved by the UCL research ethics committee (project ID − 4817/002). All of the family members, including the male members participating in this study gave written informed consent and underwent full ophthalmic examination, and all affected individuals were diagnosed as having bilateral congenital cataract (Figure 1). No dental examination was undertaken to complement the presenting cataract phenotype, as contact with the family was lost by the time the genetic characterization was available.

### 2.2. Whole exome sequencing (WES) and bioinformatic analyses

Genomic DNA was extracted from EDTA treated blood samples using the Nucleon II DNA Extraction Kit (Scotlab Bioscience, Strathclyde, Scotland, UK). The DNA samples were sequenced at Macrogen Europe. Exon capture and target enrichment were performed using the SureSelectXT Human All Exon V6 (Agilent, Santa Rosa, CA, USA). Paired-end sequencing was performed on an Illumina HiSeq 2500 high-throughput sequencer, generating mean exome coverage of 50x. Raw data in FASTQ format was aligned to the UCSC Genome Browser GRCh37/hg19 human reference sequence and analysed using the Phenopolis bioinformatics platform (10). We used a tiered approach to prioritise rare coding variants using Kaviar (http://db.systemsbiology.net/kaviar/.) (11) and Genome Aggregation Database (GnomAD v2.1 http://gnomad.broadinstitute.org/.) or rare variants (GnomAD allele frequency <0.0001) (12) in all the known cataract genes (https://cat-map.wustl.edu/). The variants were then filtered using CADD score, predicted to be moderately or highly damaging (CADD > 15) with the highest at the top for both known and unknown genes for cataracts. Further, bioinformatic validations were done on the Varsome platform (https://varsome.com; version 11.9). Direct Sanger sequencing was performed to validate the variant identified by WES using *BCOR* specific primers forward (*caccccattagtgatgcgta*) and reverse primer (*tcttccgaccagcttctgtt*) designed with http://bioinfo.ut.ee/primer3–0.4.0/.

## 3. Results

**Figure 2.**
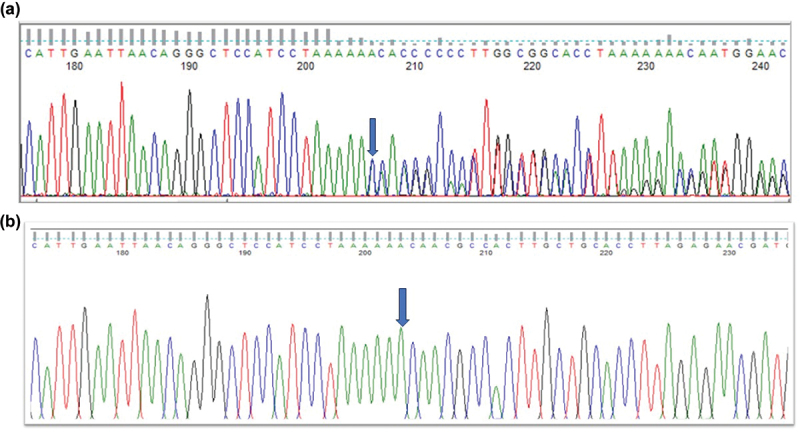
Sequence analysis - (a) *BCOR*–frameshift variant c.3621delA in affected member, (b) *BCOR* wild type in normal individual.

**Figure 3.**
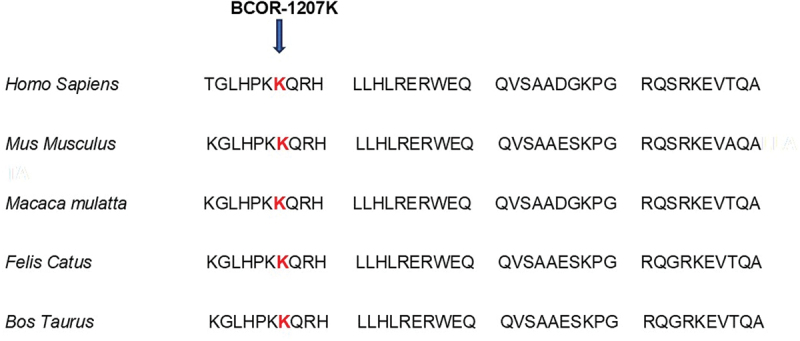
The multiple-sequence alignments from different vertebrate species. Arrows show conserved lysine at p.1207 (https://www.ncbi.nlm.nih.gov/nuccore/?term=BCOR).

A three-generation British pedigree with 4 affected, 7 unaffected, and 4 unrelated spouses presented with bilateral congenital nuclear cataract (Figure 1). All family members were examined at the slit lamp and those who were affected had bilateral nuclear cataracts. An affected DNA sample III-7 was sent for WES. After the Phenopolis genetic variant analysis pipeline, variants were filtered by allele frequency and from a total of 3036 rare coding variants, 58 rare variants remained. The top scoring variant for CADD was a rare heterozygous variant with high impact c.3621delA; p.K1207Nfs *31 in exon 8 of 15 coding the *BCOR* gene with a score of 28.9 (Table 1). Direct Sanger sequencing confirmed the variant sequence (Figure 2), which co-segregated in all the available affected family members. The conserved lysine p.1207 is mutated to asparagine, and it is followed by a 31-residue frameshifted sequence (Figure 3).

**Table 1.** Pathogenicity scores of *BCOR* variant.

Gene	Genomic pos./Exon	HGVSp	MutationTaster/verdict	GERP NR	CADD	SIFT	DANN
*BCOR*	ChrXp11.4/ Ex-8/15	p.K1207N fs*31	Disease-causing/0.81-Likely Pathogenic	5.9	28.9	Damaging	0.997

Combined Annotation Dependent Depletion (CADD) is a score for the deleteriousness of a variant. A CADD score >15 is considered damaging; Sorts Intolerant From Tolerant (SIFT) score (0.0–0.05) to check the deleteriousness of the amino acid substitution on the protein function; Genomic Evolutionary Rate Profiling (GERP) NR corresponds to the neutral rate conservation score of the site; * indicates the truncated protein; Deep Neural Network (DANN) is a score to predict the pathogenicity of the variant and ranges between 0 to 1, higher value indicates that the variant is likely to be deleterious.

## 4. Discussion

We report a truncating and frameshift variant in *BCOR* that causes nuclear cataract in females, suggesting an X-linked heterozygous inheritance in a three-generation British pedigree. BCOR is a ubiquitously expressed protein, but two dramatically different syndromes arise from pathogenic variants in *BCOR* (BCL-6 corepressor): OFCD syndrome (1, 2), which affects females, and a severe microphthalmia (“Lenz”-type) syndrome affecting primarily males. OFCD syndrome is an X-linked dominant syndrome caused by a variety of *BCOR* null mutations. As it manifests only in females, it is presumed to be lethal in males. OFCD syndrome patients have a much broader spectrum of phenotypes associated with ocular abnormalities including congenital cataracts, microphthalmia, and microcornea; dysmorphic facial features; cleft palate; cardiac abnormalities and dental anomalies (13–16). The severe male X-linked recessive microphthalmia syndrome (“Lenz”) usually includes developmental delay and is caused by hypomorphic *BCOR* variants, mainly by a specific missense variant c.254C > T, p.Pro85Leu (1, 17, 18). Given that the severe and apparent phenotype of OFCD syndrome is not included in the clinical notes of any participating family members, and no males in the family are affected, we hypothesise that this is a case of isolated congenital cataract. It is therefore clear that BCOR is likely involved in many different transcriptional networks, and the study here suggests this includes those important in eye and lens development. However, this conclusion should be taken cautiously, as dental x-ray and echocardiogram records are necessary for validation, but are unavailable for this study.

**Figure 4.**
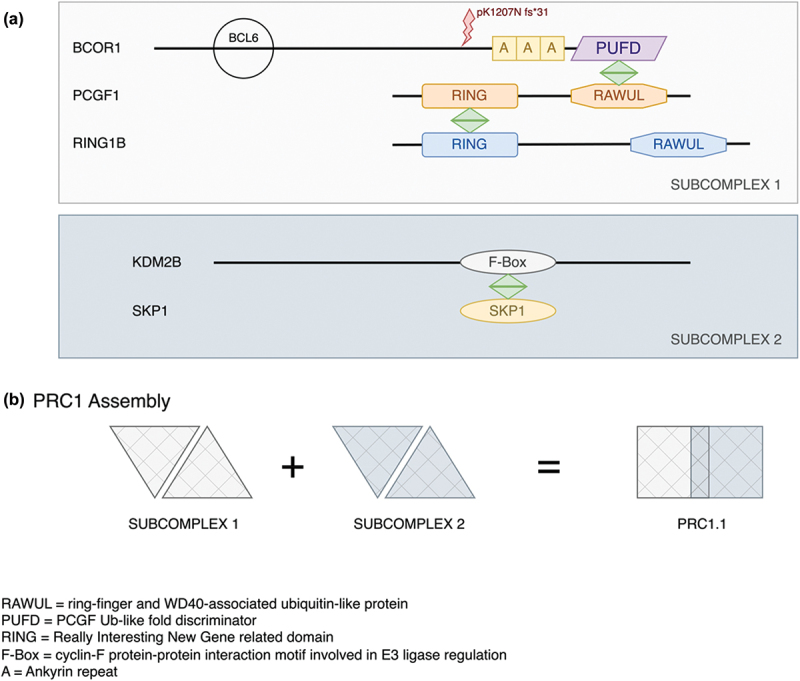
Protein interactions between BCOR1, PCGF1 and KDM2B in the PRC1.1 complex. The schematic highlights the important interactions between BCOR1, PCGF1 and KDM2B involved in the formation of the complex PRC1.1. Both the BCL2 binding site and the p.1207 frameshift mutation in *BCOR1* are indicated relative to the ankyrin repeats and the important PUFD domain. (b) The work of Wong et al. 2016 showed that BCOR1-PCGF1 dimers were required to form a hetero-tetrameric complex with the KDM2B-SKP1 dimer. PCGF1 binds RING1B to form the subcomplex 1 via the RING domains. KDM2B binds SKP1 via its F-box motif. These two subdomains combine to provide the core PRC1.1 which can then bind a wide range of accessory proteins. The p1207 frameshift mutation reported here will truncate BCOR1 before the ankyrin repeats and will therefore remove the PUFD domain needed for dimerization with PCGF1 with the result that KDMK2 will also not bind. Note the proteins and their domains are not drawn to scale.

The c.3621delA variant reported here impacts the C-terminal region of BCOR, just upstream of the ankyrin repeat region (Pfam: PF15808 https://www.ebi.ac.uk/interpro/entry/pfam/PF15808/). BCOR is a 1755 amino-acid protein (https://www.uniprot.org/uniprotkb/Q6W2J9/entry.) identified as a POZ/zinc finger transcriptional repressor (19). Within this C-terminal domain that would be removed in the c.3621delA variant, there is a PCGF1 binding site, called the PCGF Ub-like fold discriminator (PUFD) domain (Figure 4a). A complex between BCOR and PCGF1 along with the RING-domain protein RING1B is required for the binding of KDM2B along with the F-box binding protein SKP1 that constitutes the Polycomb Repressor Complex PRC1 (Figure 4) (20).

The PRC1 complex is involved in chromatin remodelling during mammalian development, and it is necessary for the correct differentiation of lens fibre cells (21–23). KDM2B, a lysine demethylase (Figure 4) is highly expressed during lens development (https://research.bioinformatics.udel.edu/iSyTE/ppi/expression.php). When KDM2B is knocked out in the developing retina, micro-ophthalmia results (24). The C-terminal-affecting mutation reported here in *BCOR* would be expected to abolish the interaction of BCOR with both KDM2B and PCGF1. This, we believe, resulted in the lens cataract phenotype in the pedigree reported here.

## References

[1] Ng D, Thakker N, Corcoran CM, Donnai D, Perveen R, Schneider A, Hadley DW, Tifft C, Zhang L, Wilkie AOM, et al. Oculofaciocardiodental and Lenz microphthalmia syndromes result from distinct classes of mutations in *BCOR*. Nat Genet. 2004;36(4):411–416. doi:10.1038/ng1321. 15004558

[2] Traboulsi EI, Lenz W, Gonzales-Ramos M, Siegel J, Macrae WG, Maumenee IH. The Lenz microphthalmia syndrome. Am J Ophthalmol. 1988;105:40–45. doi:10.1016/0002-9394(88)90119-5. 3276203

[3] François J. Genetics of cataract. Ophthalmologica. 1982;184(2):61–71. doi:10.1159/000309186. 7063172

[4] Ding X, Patel M, Herzlich AA, Sieving PC, Chan C-C. Ophthalmic pathology of nance-horan syndrome: case report and review of the literature. Ophthalmic Genet. 2009;30(3):127–135. doi:10.1080/13816810902822021. 19941417 PMC2791400

[5] Loi M. Lowe syndrome. Orphanet J Rare Dis. 2006;1(1):16. doi:10.1186/1750-1172-1-16. 16722554 PMC1526415

[6] Ionides A, Francis P, Berry V, Mackay D, Bhattacharya S, Shiels A, Moore A. Clinical and genetic heterogeneity in autosomal dominant cataract. Br J Ophthalmol. 1999;83:802–808. doi:10.1136/bjo.83.7.802. 10381667 PMC1723116

[7] Francis PJ, Berry V, Bhattacharya SS, Moore AT. The genetics of childhood cataract. J Med Genet. 2000;37:481–488. doi:10.1136/jmg.37.7.481. 10882749 PMC1734631

[8] Shiels A, Hejtmancik JF. Mutations and mechanisms in congenital and age-related cataracts. Exp Eye Res. 2017;156:95–102. doi:10.1016/j.exer.2016.06.011. 27334249 PMC5538314

[9] Berry V, Georgiou M, Fujinami K, Quinlan R, Moore A, Michaelides M. Inherited cataracts: molecular genetics, clinical features, disease mechanisms and novel therapeutic approaches. Br J Ophthalmol. 2020;104:1331–1337. doi:10.1136/bjophthalmol-2019-315282. 32217542

[10] Pontikos N, Yu J, Moghul I, Withington L, Blanco-Kelly F, Vulliamy T, Wong TLE, Murphy C, Cipriani V, Fiorentino A, et al. Phenopolis: an open platform for harmonization and analysis of genetic and phenotypic data. Bioinformatics. 2017;33(15):2421–2423. doi:10.1093/bioinformatics/btx147. 28334266

[11] Glusman G, Caballero J, Mauldin DE, Hood L, Roach JC. Kaviar: an accessible system for testing SNV novelty. Bioinformatics. 2011;27:3216–3217. doi:10.1093/bioinformatics/btr540. 21965822 PMC3208392

[12] McLaren W, Gil L, Hunt SE, Riat HS, Ritchie GRS, Thormann A, Flicek P, Cunningham F. The ensembl variant effect predictor. Genome Biol. 2016;17:122. doi:10.1186/s13059-016-0974-4. 27268795 PMC4893825

[13] Aalfs CM, Oosterwijk JC, Van Schooneveld MJ, Begeman CJ, Wabeke KB, Hennekam RCM. Cataracts, radiculomegaly, septal heart defects and hearing loss in two unrelated adult females with normal intelligence and similar facial appearance: confirmation of a syndrome? Clin Dysmorphol. 1996;5:93. doi:10.1097/00019605-199604000-00001. 8723559

[14] Davoody A, Chen I-P, Nanda R, Uribe F, Reichenberger EJ. Oculofaciocardiodental syndrome: a rare case and review of the literature. Cleft Palate Craniofac J. 2012;49:55–60. doi:10.1597/10-256. PMC3354011 21740180

[15] Feberwee HE, Feenstra I, Oberoi S, Sama IE, Ockeloen CW, Clum F, Slavotinek A, Kuijpers MAR, Dooijes D, Kuijpers‐Jagtman AM, et al. Novel BCOR mutations in patients with oculofaciocardiodental (OFCD) syndrome. Clin Genet. 2014;85(2):194–197. doi:10.1111/cge.12125. 23557072

[16] Gorlin RJ, Marashi AH, Obwegeser HL. Oculo-facio-cardio-dental (OFCD) syndrome. Am J Med Genet. 1996;63(1):290–292. doi:10.1002/(SICI)1096-8628(19960503)63:1<290:AID-AJMG47>3.0.CO;2-G. 8723122

[17] Hilton E, Johnston J, Whalen S, Okamoto N, Hatsukawa Y, Nishio J, Kohara H, Hirano Y, Mizuno S, Torii C, et al. BCOR analysis in patients with OFCD and Lenz microphthalmia syndromes, mental retardation with ocular anomalies, and cardiac laterality defects. Eur J Hum Genet. 2009;17(10):1325–1335. doi:10.1038/ejhg.2009.52. 19367324 PMC2826145

[18] Suzumori N, Kaname T, Muramatsu Y, Yanagi K, Kumagai K, Mizuno S, Naritomi K, Saitoh S, Sugiura‐Ogasawara M. Prenatal diagnosis of X-linked recessive Lenz microphthalmia syndrome. J Obstet Gynaecol. 2013;39:1545–1547. doi:10.1111/jog.12081. 23815237

[19] Huynh KD, Fischle W, Verdin E, Bardwell VJ. BCoR, a novel corepressor involved in BCL-6 repression. Genes Dev. 2000;14:1810–1823. doi:10.1101/gad.14.14.1810. 10898795 PMC316791

[20] Wong SJ, Gearhart MD, Taylor AB, Nanyes D, Ha D, Robinson A, Artigas J, Lee O, Demeler B, Hart P, et al. KDM2B recruitment of the polycomb group complex, PRC1.1, requires cooperation between PCGF1 and BCORL1. Structure. 2016;24(10):1795–1801. doi:10.1016/j.str.2016.07.011. 27568929 PMC5088048

[21] Macrae TA, Fothergill-Robinson J, Ramalho-Santos M. Regulation, functions and transmission of bivalent chromatin during mammalian development. Nat Rev Mol Cell Biol. 2023;24(1):6–26. doi:10.1038/s41580-022-00518-2. 36028557

[22] Limi S, Senecal A, Coleman R, Lopez-Jones M, Guo P, Polumbo C, Singer RH, Skoultchi AI, Cvekl A. Transcriptional burst fraction and size dynamics during lens fiber cell differentiation and detailed insights into the denucleation process. J Biol Chem. 2018;293:13176–13190. doi:10.1074/jbc.RA118.001927. 29959226 PMC6109918

[23] He S, Limi S, McGreal RS, Xie Q, Brennan LA, Kantorow WL, Kokavec J, Majumdar R, Hou H, Edelmann W, et al. Chromatin remodeling enzyme Snf2h regulates embryonic lens differentiation and denucleation. Development. 2016;143(11):1937–1947. doi:10.1242/dev.135285. 27246713 PMC4920164

[24] Iwagawa T, Fukushima M, Takeuchi S, Kawamura Y, Aihara Y, Ozawa M, Yakushiji‐Kaminatsui N, Aihara M, Koseki H, Suzuki Y, et al. The histone H3K36 demethylase Fbxl11 plays pivotal roles in the development of retinal late-born cell types. Genes Cells. 2023;28(7):482–495. doi:10.1111/gtc.13028. 37073980

